# The Other Face of *Stenotrophomonas maltophilia* in Hospitalized Patients: Insights from over Two Decades of Non-Cystic Fibrosis Cohort

**DOI:** 10.3390/antibiotics15010042

**Published:** 2026-01-01

**Authors:** Marwan Jabr Alwazzeh, Amani Alnimr, Sara M. Alwarthan, Mashael Alhajri, Jumanah Algazaq, Bashayer M. AlShehail, Abdullah H. Alnasser, Ali Tahir Alwail, Komail Mohammed Alramadhan, Abdullah Yousef Alramadan, Faisal Abdulaziz Almulhim, Ghayah Ahmed Almulhim, Jawad ur Rahman, Mohammad Taha Al-Hariri

**Affiliations:** 1 Infectious Disease Division, Department of Internal Medicine, Faculty of Medicine, Imam Abdulrahman Bin Faisal University, Dammam 31441, Saudi Arabia; smalwarthan@iau.edu.sa (S.M.A.); mhajri@iau.edu.sa (M.A.); jnalqazaq@iau.edu.sa (J.A.); ghayah.a.m@gmail.com (G.A.A.); 2 King Fahad Hospital of the University, Al Khobar 31952, Saudi Arabia; abdullah_alnasir@hotmail.com (A.H.A.); alialwayel7@gmail.com (A.T.A.); kmmhr2@gmail.com (K.M.A.); abdullah.y.alramadan@gmail.com (A.Y.A.); almolhim99@gmail.com (F.A.A.); 3 Department of Microbiology, King Fahad Hospital of the University, College of Medicine, Imam Abdulrahman Bin Faisal University, Dammam 31441, Saudi Arabia; amalnimr@iau.edu.sa; 4 Pharmacy Practice Department, College of Clinical Pharmacy, Imam Abdulrahman Bin Faisal University, Dammam 31441, Saudi Arabia; bmalshehail@iau.edu.sa; 5 Department of Internal Medicine, Faculty of Medicine, Imam Abdulrahman Bin Faisal University, Dammam 31441, Saudi Arabia; 6 Department of Microbiology, College of Medicine, Imam Abdulrahman Bin Faisal University, Dammam 31441, Saudi Arabia; juurrahman@iau.edu.sa; 7 Department of Physiology, College of Medicine, Imam Abdulrahman Bin Faisal University, Dammam 31441, Saudi Arabia; mtalhariri@iau.edu.sa

**Keywords:** antibiotic resistance, hospital-acquired infections, mortality, outcomes, *Stenotrophomonas maltophilia*, susceptibility-guided therapy

## Abstract

**Background:** *Stenotrophomonas maltophilia* is an intrinsically multidrug-resistant, biofilm- forming, non-fermenter increasingly implicated in hospital-acquired infections. Evidence from non-cystic fibrosis populations, especially in the Middle East, remains sparse. **Methods:** We conducted a retrospective observational cohort study at a tertiary academic center (Al-Khobar, Saudi Arabia) spanning 1 May 2001–30 April 2023. Hospitalized adults (≥18 years) with culture-confirmed, clinically diagnosed *S. maltophilia* infection and ≥72 h of antibiotic therapy were included. The primary outcome was all-cause mortality (14-day, 30-day, 1-year). Secondary outcomes were clinical response, microbiological eradication, and infection recurrence. Predictors of 30-day mortality were assessed using multivariable logistic regression; 14-day mortality was analyzed by Kaplan–Meier/log-rank according to susceptibility-guided versus alternative therapy. **Results:** Of 539 patients with positive cultures, 436 met the inclusion criteria. Mean age was 60.5 ± 19.3 years; 62.2% were male. Most infections were hospital-acquired (92.9%); pneumonia composed 64.7% and bloodstream infection 15.4%. Polymicrobial growth occurred in 55.5% (predominantly Gram-negative co-isolation). Susceptibility was 95.1% to trimethoprim–sulfamethoxazole, 76.4% to levofloxacin, and 43.6% to ceftazidime. Mortality at 14 days, 30 days, and 1 year was 22.8%, 37.9%, and 57.2%, respectively. On multivariable modelling, intensive care unit (ICU) admission, leukocytosis, neutrophilia, anemia, and thrombocytopenia independently predicted 30-day mortality. Susceptibility-guided therapy was associated with improved 14-day survival (log-rank *p* = 0.033). **Conclusions:** In this large, long-running non-cystic fibrosis cohort, host acuity and early alignment of treatment to susceptibility data were dominant drivers of outcome. High polymicrobial burden and limited reliably active agents underscore the need for meticulous stewardship, robust infection prevention, and cautious interpretation of *S. maltophilia* antimicrobial susceptibility testing.

## 1. Introduction

*Stenotrophomonas maltophilia* is an aerobic, non-spore-forming, Gram-negative bacillus [1]. It is a widespread environmental pathogen recovered from diverse natural habitats, including surface waters like rivers and lakes, soil, plants, animals, food, as well as healthcare environments [1,2]. Its ability to persist in moist, nutrient-scarce environments, including disinfectant solutions and medical equipment such as ventilators and intravascular catheters, makes it a formidable nosocomial pathogen, and early ecological studies further demonstrated its survival in hospital water-associated niches like sink traps and faucet aerators, underscoring its opportunistic role in healthcare settings [3]. It was long regarded as an opportunistic bacterium with low virulence, primarily affecting immunocompromised patients, including those with cystic fibrosis (CF) or malignancies. Additionally, difficulty in distinguishing between *S. maltophilia* colonization and infection in routine practice has contributed to the perception that *S. maltophilia* is a pathogen with limited clinical significance [4]. While the role of *S. maltophilia* in CF patients has been widely studied, the burden in non-CF inpatients is less well characterized, despite its rising clinical significance. Recent hospital epidemiology demonstrates a rising incidence of *S. maltophilia* infections in both community-acquired and nosocomial settings [5,6]. This increasing prevalence is strongly associated with broader healthcare exposures, such as prolonged hospitalization, indwelling device use, and prior antibiotic therapy [7]. Ventilator-associated pneumonia (VAP), bloodstream infections (BSIs), and device-related sepsis are the most common human *S. maltophilia* infections reported [8,9]. Other documented *S. maltophilia* infections include skin and soft tissue infections, urinary tract infections, endocarditis, central nervous system infections, ophthalmologic infections, osteomyelitis, and gastrointestinal infections [2,4].

Although *S. maltophilia* is often labelled a low-virulence pathogen [10], the accumulated evidence suggests that *S. maltophilia* infections pose significant therapeutic challenges and are associated with considerable morbidity and elevated mortality rates, reflecting both the intrinsic resistance mechanisms of the organism and the vulnerability of affected populations [9]. Proposed virulence factors include motility, biofilm formation, production of extracellular enzymes, and iron acquisition mechanisms [2].

*S. maltophilia* exhibits multiple antimicrobial resistance mechanisms, making it resistant to most β-lactams, including carbapenems, as well as polymyxins, fluoroquinolones, aminoglycosides, tetracyclines, and macrolides. These mechanisms can be categorized as intrinsic, acquired, or phenotypic. The primary intrinsic resistance mechanisms include chromosomally encoded efflux pumps, such as the SmeDEF system; antibiotic-inactivating enzymes, especially β-lactamases (notably the L1 metallo-β-lactamase and L2 serine-β-lactamase); aminoglycoside-modifying enzymes, and decreased outer membrane permeability [5,9,11]. Acquired resistance emerges via horizontal gene transfer or regulatory mutations that upregulate intrinsic determinants [11,12]; prominent examples include Qnr determinants, which protect DNA from fluoroquinolone inhibition, metallo-β-lactamases, and class 1 integrons [5,9]. Additionally, biofilm-associated phenotypic resistance is increasingly recognized [12]. Notably, efflux over-expression selected by trimethoprim–sulfamethoxazole (TMP-SMX), fluoroquinolones, or tigecycline can generate cross-resistance [11]. Resistance to various antibiotic classes is increasing, with a multidrug-resistant profile severely limiting reliable therapeutic options, underscoring the importance of TMP-SMX as the cornerstone of therapy [13].

Over the past decade, iterative changes in Clinical and Laboratory Standards Institute (CLSI) breakpoints for *S. maltophilia* have mirrored growing uncertainty around the reliability of antimicrobial susceptibility testing. Ceftazidime interpretive criteria were ultimately withdrawn due to intrinsic β-lactamase activity and poor test reproducibility; levofloxacin was retained with stewardship cautions against monotherapy; TMP-SMX remained a reference agent, typically first-line when susceptible. The challenges in Antimicrobial susceptibility testing (AST) have led to a reliance on dual combination therapies of TMP-SMX, levofloxacin, cefiderocol, or minocycline, or the ceftazidime-avibactam/aztreonam combination, despite a lack of robust clinical trial evidence to support this practice [14,15].

As the antibiotic resistance dilemma escalates globally, *S. maltophilia* should not be overlooked as a potential emerging superbug in high-acuity, device-dense environments. Understanding the epidemiology, risk factors, and treatment outcomes in non-CF patients is therefore essential to optimize management strategies in real-world hospital practice.

This study aims to contribute to the limited literature on *S. maltophilia* infections by providing new insights into clinical features and microbiological characteristics, examining the status of acquired resistance to the limited antibiotic therapy options, and evaluating management outcomes and mortality predictors in a cohort of non-CF hospitalized patients treated over more than two decades at a tertiary care center. With its long-term, large-scale cohort design, this study offers valuable insights into *S. maltophilia* infections in non-CF hospitalized patients from the Middle East.

## 2. Results

### 2.1. Demographic and Clinical Data

**Figure 1:**
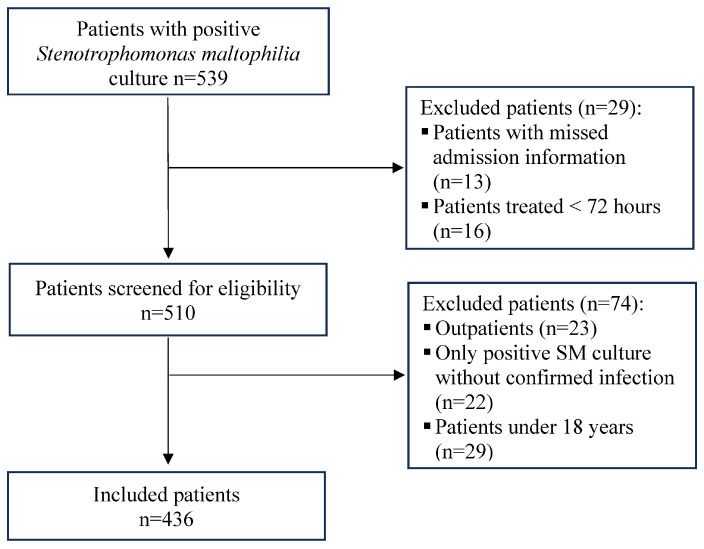
Study flowchart of *Stenotrophomonas maltophilia* infections in hospitalized patients.

Out of 539 patients with positive *S. maltophilia* cultures, 436 had confirmed *S. maltophilia* infections that met the inclusion criteria (Figure 1). Two hundred seventy-one patients (62.2%) were males, and the average age was 60.53 ± 19.31 years. Most *S. maltophilia* infections were hospital-acquired (92.9%). The main comorbidities identified were diabetes mellitus (DM) (39.0%) and renal disease (22.0%). The documented *S. maltophilia* infections showed a clear upward trend over the study period, increasing from 18 cases before 2004 to 35 during 2004–2007, then to 73 in 2008–2011, and reaching 105 in 2012–2015. The incidence plateaued at 101 cases during 2016–2019 and remained stable during the COVID-19 pandemic period (2020–2023) with 104 cases.

Hospital-acquired pneumonia (HAP), ventilator-associated pneumonia (VAP), and bloodstream infections (BSIs) were the most commonly occurring *S. maltophilia* infections, accounting for 31.0%, 27.3%, and 15.4% of cases, respectively. Other infection types included skin and soft tissue infections (7.8%), urinary tract infections (UTIs) (6.2%), and wound infections (5.3%). A small proportion presented with ear infections (2.1%).

Hospital-acquired infections, renal failure, central nervous system infections, admission to an ICU, central venous catheter indwelling, mechanical ventilation, and prior antibiotic therapy within the last three months were significantly linked to 30-day mortality (Table 1). The mean time of hospitalization until the first positive *S. maltophilia* culture was 29.4 ± 54.7 days.

As shown in Table 2, non-survivors at day 30 post-*S. maltophilia* infection had significantly higher neutrophil counts, C-reactive protein, aspartate aminotransferase, blood urea nitrogen (BUN), and longer prothrombin and partial thromboplastin times (PT and aPTT). Platelet counts, hemoglobin, and total protein levels were significantly decreased.

### 2.2. Microbiological Data

**Figure 2:**
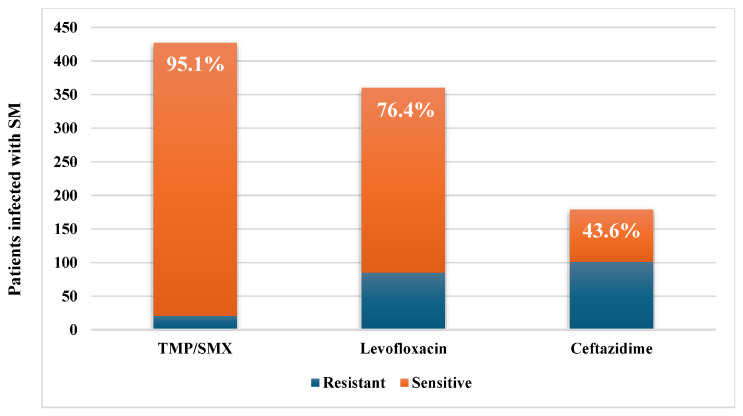
Susceptibility to TMP-SMX, levofloxacin, and ceftazidime was isolated from samples taken from hospitalized patients with *S. maltophilia* infections.

In 55.5% of cases, other concomitant microbes were isolated, mainly Gram-negative bacteria (79.5%), with a predominance of *Pseudomonas aeruginosa*, *Acinetobacter baumannii*, and *Klebsiella pneumoniae*. Additionally, Gram-positive bacteria and Candida species were isolated from 12.2% and 8.3% of patients, respectively (Table 3). Susceptibility rates to the three commonly used agents are shown in Figure 2; resistance rates were 56.4% to ceftazidime and 23.6% to levofloxacin, with TMP-SMX remaining active in 95.1%.

### 2.3. Outcomes and Mortality Predictors

**Figure 3:**
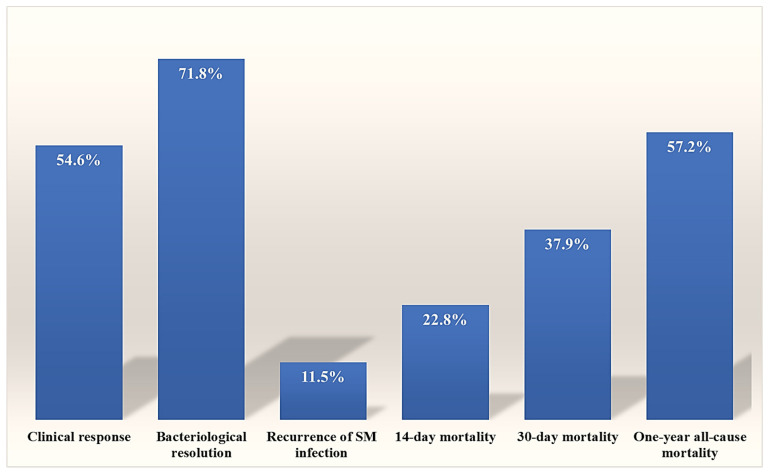
Primary and secondary outcomes of hospitalized patients with *S. maltophilia* infections.

Regarding the primary outcomes, 14-day, 30-day, and one-year overall mortality rates were 22.8%, 37.9%, and 57.2%, respectively. Notably, the 30-day mortality rate was significantly higher during the COVID-19 pandemic period compared to 2016–2019 (41 patients, 39.0% vs. 23 patients, 22.8%; *p* = 0.012). The secondary outcomes showed clinical response in 54.6% of cases, bacteriological eradication in 71.8%, while the recurrence rate of *S. maltophilia* infections was 11.5% (Figure 3). The recurrence of *S. maltophilia* infections was significantly higher in patients with polymicrobial infections (*p* = 0.002).

**Figure 4:**
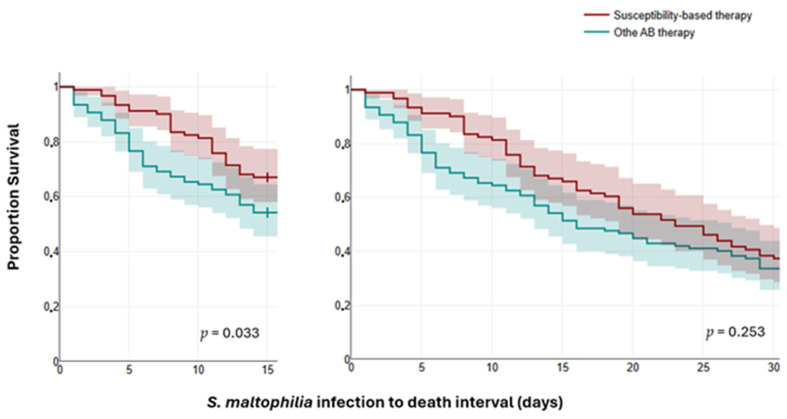
Kaplan–Meier curves show 14-day- and 30-day mortality in hospitalized patients treated with susceptibility-based therapy vs. other AB therapy options.

The univariable and multivariable logistic regression analyses indicate that high leukocyte counts, high neutrophil percentage, low hemoglobin levels, low platelet counts, and ICU admission were independent predictors of 30-day mortality (Table 4 and Table 5). In addition, a logistic regression model included DM, renal failure, previous hospitalization, prior antibiotic therapy, ICU admission, hospital-acquired *S. maltophilia* infections, ventilator-associated pneumonia, central venous catheter indwelling, mechanical ventilation, and receiving antibiotic monotherapy (TMP-SMX, levofloxacin, or ceftazidime) indicates that previous hospitalization within the past three months (adjusted odds ratio [OR], 0.404; 95% confidence interval [CI], 0.171–0.955; *p* = 0.039) and previous antibiotic therapy within the past three months (adjusted OR, 0.430; 95% CI, 0.195–0.947; *p* = 0.036) were independent predictors of lack of clinical response. In addition, previous hospitalization within the past three months was an independent predictor of *S. maltophilia* infection recurrence (adjusted OR, 3.016; 95% CI, 1.243–7.314; *p* = 0.015). Furthermore, survival analysis indicated that susceptibility-based therapy was significantly correlated with reduced 14-day mortality compared to other antibiotic therapies (Figure 4).

## 3. Discussion

In light of the escalating global threat of antimicrobial resistance, it is essential not to overlook the significance of *S. maltophilia* as an intrinsically multidrug-resistant pathogen. Despite historical perceptions that *S. maltophilia* is an opportunistic, low-virulence pathogen [10], emerging data indicate that *S. maltophilia* infections are associated with significant morbidity and high mortality [7,16]. In addition, *S. maltophilia* infections pose substantial therapeutic challenges, with a notable increase in reported cases among both hospitalized patients and the general population [5,17]. 

This 22-year, single-center, non-CF cohort study found that most patients (92.9%) developed hospital-acquired infections with a device- and ventilation-heavy ecology, while 7.1% were diagnosed with *S. maltophilia* infection within 48 h of admission and had no known prior healthcare exposure, suggesting a community source. These findings align with previous reports emphasizing the predominance of healthcare-associated *S. maltophilia* infections; however, some studies have reported higher rates of community-acquired cases [18,19]. During the study period, *S. maltophilia* infection incidence increased threefold from 2004–2007 to 2012–2015, then plateaued, aligning with previously reported increased incidence in Saudi Arabia [6].

Previous studies rarely reported the length of hospitalization before *S. maltophilia* infection. In our study, the average duration was 29.4 ± 54.7 days, with a median of 15 days, which is shorter than the 19 days reported by Insuwanno et al. [20].

More than half of patients received antibiotic therapy within the past three months, aligning with previous studies that identified the use of broad-spectrum antibiotics, especially carbapenems, as a risk factor for *S. maltophilia* infections [21,22,23]. Other documented risk factors include prolonged hospitalization, impaired immune status, severe illness, high Sequential Organ Failure Assessment (SOFA) score, indwelling central catheter, and mechanical ventilation [23,24].

In our cohort, pneumonia was the most frequent type of *S. maltophilia* infections (60.6%), particularly HAP and VAP, followed by BSI (15.4%), consistent with previous studies [16,25]. Other observed types include skin and soft tissue infections, UTIs, wound infections, and intra-abdominal infections. In addition, ear infections were documented in nine patients (2.1%), a finding rarely reported in the literature.

Regarding microbiological characteristics, polymicrobial isolates were frequent (55.5%). Most co-isolates were Gram-negative bacteria (79.5%), predominantly *Pseudomonas aeruginosa* and *Acinetobacter baumannii*. These findings are consistent with earlier research indicating that *S. maltophilia* is frequently isolated alongside other healthcare-associated pathogens, including *Pseudomonas aeruginosa*, various *Enterobacteriaceae*, *Staphylococci*, and fungal species [2,26,27]. However, lower rates of bloodstream polymicrobial infections were observed [28].

Polymicrobial cultures pose challenges, making it difficult to determine the role of *S. maltophilia*, thereby delaying microbiological diagnosis and complicating treatment decisions. Additionally, the coexistence of microbes can result in polymicrobial interactions that facilitate the exchange of resistance genes and influence the virulence of the involved pathogens [26].

Susceptibility profiles in our cohort were characterized by sustained TMP-SMX activity, moderate levofloxacin susceptibility, and poor ceftazidime activity, echoing contemporary concerns about β-lactamase-mediated resistance and variable test performance. The high susceptibility to TMP-SMX observed here was also reported in previous studies, with rates ranging from 88.7% to 98.1% [18,24,29,30]. However, about 25% of isolates were resistant to levofloxacin, which contrasts with earlier reported susceptibility rates between 88% and 96.6% [24,29,30]. The high levofloxacin resistance rate in our cohort may indicate the overuse of fluoroquinolones, which are not restricted under the institution’s antibiotic prescribing policy. Additionally, some researchers warn that resistance to fluoroquinolones may develop during therapy with levofloxacin [30]. Furthermore, the high rate of resistance to ceftazidime found in our study aligns with previous reports [20]. These findings highlight the alarming rise of resistance against the few remaining antibiotic options to combat *S. maltophilia*. On the other hand, given breakpoint volatility, methodological limitations, and frequent CLSI adjustments, including withdrawal of ceftazidime breakpoints, *S. maltophilia* AST warrants cautious interpretation and clinical correlation [31].

As shown in Table 3, there were no statistically significant differences in 30-day mortality between patients receiving monotherapy and those receiving combination therapy. These results align with the study by Appaneal et al., which involved 3891 hospitalized patients. They found similar clinical outcomes regardless of whether the *S. maltophilia* isolates were multidrug-resistant, resistant to TMP-SMX or levofloxacin, or non-resistant [32]. Additionally, Shah et al. concluded that there are no differences in clinical outcomes whether the patient receives TMP-SMX or a fluoroquinolone as monotherapy [33]. Furthermore, our study found no significant difference in 30-day mortality between susceptibility-based antibiotic therapy and other antibiotic treatment options. In contrast, as shown in Figure 3, susceptibility-based therapy significantly reduced the 14-day mortality rate (*p* = 0.033), whether mono- or combination susceptibility-based therapy was used. However, mortality in critically ill patients is usually affected by multiple factors; 14-day mortality is likely more influenced by *S. maltophilia* infections and the applied antimicrobial therapy rather than 30-day mortality. These findings align with previous studies reporting a decrease in 14-day mortality among infected patients who receive timely, appropriate antibiotic therapy [29,34].

Taken together, early alignment of therapy to likely susceptibility, rather than reflex escalation or routine combination, appears most impactful in the short term, particularly in ICU contexts with high device/biofilm burden. Despite evolving CLSI guidance (2013–2025), the short-term benefit of susceptibility-guided therapy persisted in our data, reinforcing tailored treatment in complex, polymicrobial settings [35,36]. Newer options (e.g., cefiderocol, aztreonam with avibactam, or ceftazidime–avibactam) show promising in vitro activity but limited clinical evidence and were unused in our period of observation.

In terms of the primary outcomes, 14-day mortality, which more accurately reflects the impact of *S. maltophilia* infection, is rarely reported. Kanchanasuwan et al. reported a comparable 14-day mortality rate of 21.4% [29], while reported 30-day mortality rates ranged from 29.1% to 36.1%, aligning more closely with the rate found in our study [21,29,37]. However, a higher 30-day mortality rate of 54.3% was observed in infected patients after allogeneic hematopoietic stem cell transplantation [25]. Remarkably, our cohort showed a significantly higher 30-day mortality rate during the COVID-19 pandemic compared to the 2016–2019 period, which supports Raad et al.’s findings in critical COVID-19 patients with *S. maltophilia* pneumonia [38]. The one-year all-cause mortality rate has not been previously reported; however, in-hospital mortality rates reported earlier ranged from 30.7% to 56.0% [7,16,20,29]. In their review of 19 studies, Huang et al. estimated the mortality rate of *S. maltophilia*-infected patients to be 40.5% [39]. The pattern suggests host acuity, rather than organism “virulence” alone, is the dominant driver. Independent predictors (ICU admission, leukocytosis, neutrophilia, anemia, thrombocytopenia) reflect systemic severity and dysregulated host response.

Regarding the 30-day mortality predictors, our findings show that mortality was influenced mainly by critical care context; hospital-acquired infections, pneumonia—especially VAP—renal failure, previous antibiotic therapy within the past three months, ICU admission, central venous catheterization, mechanical ventilation, leukocytosis, neutrophilia, anemia, thrombocytopenia, prolonged PT and aPTT, low total serum protein, high aspartate transferase, and elevated BUN. The multivariable logistic regression analysis indicates that ICU admission, leukocytosis, neutrophilia, anemia, and thrombocytopenia are independent predictors of 30-day mortality. Most of the identified mortality predictors were also reported in the previous studies [18,22,34,39,40,41]. Other identified predictors (or risk factors) in the literature include advanced age, septic shock, hematological malignancy, hypoalbuminemia, neutropenia, high bilirubin levels, receiving immunosuppressive therapy, high Charlson comorbidity index, high SOFA score, high Acute Physiological Assessment and Chronic Health Evaluation (APACHE) score, infections with *S. maltophilia* strains resistant to fluoroquinolones, and polymicrobial infections [2,20,21,39,41,42,43]. In addition, delayed or inappropriate antibiotic therapy, which was a 14-day mortality predictor in our study, has also been previously reported as a risk factor for mortality [29,34,39].

With respect to secondary outcomes, the clinical response in our population was relatively low at 54.6%, likely reflecting polymicrobial disease and non-optimal initial therapy in a subset. In comparison, previous reports showed higher clinical response rates of 76.3.0% to 87% in patients with bloodstream infection or pneumonia [33,44,45]. However, a lower clinical success rate in critically ill patients was also documented [46]. Prior hospitalization and receiving antibiotic therapy within the past three months were independent predictors of less successful clinical response in our study. Limited data on microbiological resolution and recurrence of *S. maltophilia* infections have been previously published; Nys et al. reported microbiological eradication rates of 82%, which exceeds our 71.8% [47]. In relation to the recurrence, our data showed an 11.5% rate and identified the previous hospitalization within the past three months and polymicrobial infections as independent risk factors for recurrence. A lower recurrence rate of 5.5% was reported in patients with *S. maltophilia* bloodstream infection [33]. However, a higher rate of 35.6% was reported in patients with pneumonia treated with minocycline [45]. Additionally, the recurrence rates of clinical and microbiological chest infections were 25.3% and 39.7%, respectively, observed in patients after lung transplantation [48]. The variation in documented infection recurrence rates mainly reflects the diversity of publications studied, underscoring heterogeneity in risk, exposure, and follow-up. Notably, in our cohort, the recurrence rate was significantly higher in patients with initial polymicrobial infections; however, such associations were not observed with other primary and secondary outcomes.

This study demonstrates important strengths, including the long observation horizon, a large non-CF inpatient sample, consistent operational definitions, and direct microbiology-to-outcome linkage. Key limitations, typical of real-world *S. maltophilia* research, are its retrospective, single-center nature, the high rate of polymicrobial infections that might significantly influence the study outcomes and conclusions; however, the prevalence of polymicrobial infections among the studied populations and the important role of *S. maltophilia* in such infections have been frequently demonstrated in previous studies. In addition, the uncertainty regarding susceptibility methods and breakpoint interpretations also warrants caution, and the resistance rates should be contextualized within local institutional pathways and infection-control policies, considering recent CLSI updates. Furthermore, as a retrospective study, we were unable to perform molecular characterization of resistance mechanisms (e.g., sul and qnr genes) and clonality analysis, which are critical for understanding the local epidemiology and transmission dynamics of resistant *S. maltophilia* strains. Moreover, being a single-center study limits the generalizability of the findings to other local, national, and international settings. 

## 4. Materials and Methods

### 4.1. Study Design, Settings, and Participants

We conducted a single-center retrospective observational cohort study at King Fahad Hospital of the University (KFHU), a tertiary academic medical center with over 500 beds in Al-Khobar, Eastern Province, Saudi Arabia. Medical records of hospitalized patients with culture-confirmed *S. maltophilia* infections were thoroughly reviewed from 1 May 2001 to 30 April 2023. All included cases originated from routine care, and the hospital’s electronic and paper-based records during the study period provided detailed information necessary for consistent data extraction.

Inclusion criteria were:

1. Adult ≥ 18.
2. Clinically diagnosed *S. maltophilia* infection (not colonization).
3. Receipt of directed antimicrobial therapy ≥ 72 h.

Patients were excluded if:

1. The key admission data were missing.
2. They were managed as outpatients.

The primary clinical outcome evaluated was mortality, with rates assessed at 14 days, 30 days, and one year. Secondary outcomes encompassed clinical cure, bacteriological eradication, and recurrence. Additionally, factors associated with mortality and the secondary outcomes were analyzed. Operational definitions outlined in the study protocol were applied consistently across the dataset. These included defining HAP, VAP, and BSI according to established CDC/NHSN criteria. Clinical response was defined as the resolution of symptoms and signs, along with significant improvement in inflammatory markers within 14 days of starting therapy. Bacteriologic eradication was defined as negative follow-up bacterial cultures within 14 days, and infection recurrence was considered when a new episode of *S. maltophilia* infection, regardless of infection type, was diagnosed within three months after the initial infection.

### 4.2. Demographics and Clinical Data Collection

Hospitalized patients with documented *S. maltophilia* infections were identified by electronic screening records. Paper and electronic records were abstracted into a piloted case-report form. The information collected included age, gender, comorbidities, date of hospital admission and date of index infection, acquisition (community- or hospital-acquired), infection site, baseline routine laboratory results, recent healthcare exposures (prior hospitalization/antibiotics within past three months), invasive supports (ICU, ventilation, central line), co-isolates, AST results to TMP-SMX, levofloxacin, and ceftazidime, antibiotic regimens, and subsequent primary and secondary outcomes. All available demographic and clinical information was recorded as documented in the patient files, without imputation or extrapolation. When both electronic and paper records existed for the same patient, data were cross-checked to ensure accuracy. The data collection form was used to maintain consistency in the type and sequence of variables extracted, and each variable was defined clearly to avoid ambiguity during abstraction. The focus was to capture the clinical context surrounding each infection episode and the circumstances that may have influenced outcomes. In addition, multivariable regression analysis was performed to reduce confounding bias and improve the validity of estimated associations between predictors/risk factors and outcomes.

### 4.3. Microbiological Procedures

Specimens were processed in accordance with CLSI standards. Bacterial identification protocols evolved during the study period: From 2013 to 2017, isolates were identified using the Vitek 2 system (bioMérieux, Marcy-I’Étoile, France). Starting in 2018, identification was routinely performed using matrix-assisted laser desorption ionization time-of-flight mass spectrometry (MALDI-TOF MS; bioMérieux VITEK MS) for improved accuracy in speciating non-fermenting Gram-negative bacilli. In contrast to the identification methods, the primary AST methodology remained consistent throughout the entire study period, ensuring longitudinal comparability. AST was carried out for all isolates using the automated Vitek 2 system with GN AST cards. The specific panel of antimicrobial agents reported (TMP-SMX, levofloxacin, and ceftazidime) was selected to reflect the hospital’s drug formulary and local stewardship policies. Interpretations were based on annually updated CLSI breakpoints (M100, CLSI, annually updated) [14]. Given recognized AST challenges associated with *S. maltophilia*, supplementary testing via gradient diffusion (E-test) or disc diffusion was not performed on all isolates; rather, it was reserved for verifying discrepant or indeterminate results for these formulary agents. This verification protocol was consistently applied across the study years, in accordance with the laboratory’s established internal standard operating procedures. Where CLSI interpretive changes occurred over time, contemporaneous breakpoints were applied with sensitivity analyses as appropriate. The study relied exclusively on standard, validated, and accredited processes that were part of routine patient care.

### 4.4. Statistical Analysis

Statistical analyses were performed in IBM SPSS Statistics version 26 (Armonk, NY, USA) and Jeffreys’s Amazing Statistics Program (JASP) Version 0.95.4. Normality was assessed by Shapiro–Wilk; appropriate parametric/non-parametric tests were applied; significance was two-tailed at *p* < 0.05. Group comparisons for 14- and 30-day mortality were conducted using independent t-tests for continuous data and Chi-square tests for categorical variables. Univariable and multivariable logistic regression analyses were performed after testing for multicollinearity to identify predictors of 30-day mortality and other secondary outcomes, reporting odds ratios (ORs) and adjusted odds ratios (aOR) with 95% confidence intervals. Multivariable models incorporated clinically relevant covariates to mitigate confounding and avoid over-fitting. Survival analysis for 14-day and 30-day mortality used the Kaplan–Meier method with a log-rank test comparing susceptibility-guided versus alternative therapy. Missingness was handled by complete-case analysis; the extent and pattern of missing data were reviewed and were not systematically associated with outcomes. All analyses reflected the available dataset without generating additional variables or modifying existing values. Statistical methods were selected to match the structure of the collected data and to provide a transparent interpretation of clinical and microbiological relationships within the cohort. Each analytical step was performed using the same criteria across all included patients to maintain consistency.

## 5. Conclusions

This study represents the largest and longest non-CF dataset for *S. maltophilia* in the region, underscoring that host acuity and early alignment of therapy to susceptibility data remain the most decisive factors influencing outcomes in our settings. It identifies various infection types, clinical features, microbiological characteristics, and resistance rates to three antibiotics commonly used to treat *S. maltophilia* infections. Additionally, it highlights management outcomes, including mortality rates and their predictors, clinical response, microbiological resolution, and recurrence of *S. maltophilia* infections. Further research is necessary, especially studies that focus on molecular aspects, such as characterizing resistance mechanisms and prospectively evaluating the effectiveness of newly proposed antibiotic treatment options.

## Figures and Tables

**Table 1:** Baseline demographic, clinical characteristics, and 30-day mortality risk factors of included patients with *S. maltophilia* infections (30-day survivors vs. non-survivors).

	Total (N = 436)	Survivors (N = 305)	Non-Survivors (N = 131)	*p*-Value
	n (%)	n (%)	n (%)	
**Age** (in years) *	60.53 ± 19.31	59.96 ± 20.13	61.85 ± 17.24	0.350
**Gender** (male)	271 (62.2)	195 (63.9)	76 (58.0)	0.234
**Comorbidities**				
Diabetes mellitus	170 (39.0)	114 (37.4)	56 (42.7)	0.292
Chronic pulmonary disease	9 (2.1)	7 (2.3)	2 (1.5)	0.605
Renal failure	96 (22.0)	58 (19.0)	38 (29.0)	0.021
Malignancy	24 (5.5)	16 (5.2)	8 (6.1)	0.724
**Site of infection acquisition**				
Hospital-acquired	405 (92.9)	277 (90.8)	128 (97.7)	0.010
Community-acquired	31 (7.1)	28 (9.2)	3 (2.3)	
**Infection type**				
Bloodstream infection	67 (15.4)	43 (14.1)	24 (18.3)	0.262
Pneumonia	264 (60.6)	177 (58.0)	87 (66.4)	0.101
Urinary tract infection	27 (6.2)	21 (6.9)	6 (4.6)	0.360
Skin and soft tissue infection	34 (7.8)	28 (9.2)	6 (4.6)	0.101
Wound infection	23 (5.3)	18 (5.9)	5 (3.8)	0.372
Intra-abdominal infection	9 (2.1)	7 (2.3)	2 (1.5)	0.605
Central nervous system infection	3 (0.7)	2 (0.7)	1 (0.8)	0.901
Ear infection	9 (2.1)	9 (3.0)	0 (0.0)	0.047
**Hospital-acquired infection type**				
Bloodstream infection	65 (14.9)	41 (13.4)	24 (18.3)	0.190
Hospital-acquired pneumonia	135 (31.0)	99 (32.5)	36 (27.5)	0.303
Ventilator-associated pneumonia	119 (27.3)	70 (23.0)	49 (37.4)	0.002
Urinary tract infection	22 (5.0)	16 (5.2)	6 (4.6)	0.771
Skin and soft tissue infection	28 (6.4)	23 (7.5)	5 (3.8)	0.146
Wound infection	22 (5.0)	17 (5.6)	5 (3.8)	0.442
Intra-abdominal infection	9 (2.1)	7 (2.3)	2 (1.5)	0.605
Central nervous system infection	3 (0.7)	2 (0.7)	1 (0.8)	0.901
Ear infection	2 (0.5)	2 (0.7)	0 (0.0)	0.353
**Risk factors**				
Previous hospitalization ^#^	151 (34.6)	100 (32.8)	51 (38.9)	0.216
Previous antibiotic therapy ^#^	241 (55.3)	159 (52.1)	82 (62.6)	0.044
ICU admission	177 (40.6)	96 (31.5)	81 (61.8)	<0.001
Central venous catheter	103 (23.6)	55 (18.0)	48 (36.6)	<0.001
Mechanical ventilation	158 (36.2)	87 (28.5)	71 (54.2)	<0.001
Concomitant infection	242 (55.5)	166 (54.4)	76 (58.0)	0.489
Length of stay before *S. maltophilia* infection *	29.40 ± 54.73	28.23 ± 57.4	32.12 ± 48.02	0.497

* Continuous variables were reported as mean ± standard deviation (SD). ^#^ within the past three months.

**Table 2:** A comparison of baseline laboratory test results in relation to 30-day mortality among survivors and non-survivors.

	Survivors (N = 305) (mean ± SD)	Non-Survivors (N = 131) (mean ± SD)	*p*-Value
**White blood cells**	11.50 ± 6.25	12.86 ± 8.25	0.068
**Neutrophils (%)**	71.97 ± 14.72	79.7 ± 13.96	<0.001
**Hemoglobin**	10.13 ± 2.30	9.12 ± 1.72	<0.001
**Platelets**	285.80 ± 147.1	180.70 ± 147.1	<0.001
**Prothrombin time**	15.47 ± 5.31	19.08 ± 10.73	<0.001
**Partial thromboplastin time**	36.30 ± 13.60	42.48 ± 24.81	0.002
**C-reactive protein**	8.85 ± 8.13	11.59 ± 7.87	0.017
**Procalcitonin**	3.12 ± 10.43	5.87 ± 12.15	0.163
**Total protein**	6.16 ± 1.53	5.58 ± 0.99	<0.001
**Albumin**	2.65 ± 0.63	2.58 ± 0.59	0.247
**Aspartate transferase**	47.60 ± 60.89	91.65 ± 289.6	0.018
**Alanine aminotransferase**	51.27 ± 64.90	65.28 ± 113.0	0.139
**Blood urea nitrogen**	30.59 ± 26.11	47.17 ± 35.65	<0.001
**Creatinine**	1.57 ± 1.90	1.93 ± 1.81	0.084

**Table 3:** Comparison of concomitant isolated microbes, *S. maltophilia* susceptibility, and antibacterial regimens used in relation to 30-day mortality among survivors and non-survivors.

	Total (N = 436)	Survivors (N = 305)	Non-Survivors (N = 131)	*p*-Value
	n	n (%)	n (%)	
**Concomitant isolated microbes**				
*Pseudomonas aeruginosa*	64	49 (16.1)	15 (11.5)	0.212
*Acinetobacter baumannii*	58	38 (12.5)	20 (15.3)	0.429
*Klebsiella pneumoniae*	47	29 (9.5)	18 (13.7)	0.191
*Escherichia coli*	13	11 (3.6)	2 (1.5)	0.242
*Serratia marcescens*	20	12 (3.9)	8 (6.1)	0.320
*Enterobacter* Species	13	11 (3.6)	2 (1.5)	0.242
Other Gram-negative	14	12 (3.9)	2 (1.5)	0.191
*Staphylococcus aureus*	14	11 (3.6)	3 (2.3)	0.475
Other Staphylococci	8	5 (1.6)	3 (2.3)	0.643
*Enterococcus* Species	13	9 (3.0)	4 (3.1)	0.954
*Candida* Species	24	15 (4.9)	9 (6.9)	0.413
**Antibacterial therapy**				
Susceptibility-based AB therapy	191	132 (43.3)	59 (45.0)	0.734
Other AB therapy	245	173 (56.7)	72 (55.0)	
TMP-SMX monotherapy	81	58 (19.0)	23 (17.6)	0.719
Levofloxacin monotherapy	49	32 (10.5)	17 (13.0)	0.451
Ceftazidime monotherapy	23	12 (3.9)	11 (8.4)	0.056
TMP-SMX-Levofloxacin	17	12 (3.9)	5 (3.8)	0.954
TMP-SMX + Ceftazidime	9	8 (2.6)	1 (0.8)	0.211
Levofloxacin + Ceftazedime	5	4 (1.3)	1 (0.8)	0.622
TMP-SMX + Levofloxacin + Ceftazidime	7	6 (2.0)	1 (0.8)	0.363
**AB therapy duration** (mean ± SD) *				
TMP-SMX	8.53 ± 5.72	9.51 ± 6.00	5.80 ± 3.73	0.002
Levofloxacin	8.24 ± 11.31	8.91 ± 13.17	6.75 ± 4.98	0.440
Ceftazidime	8.11 ± 7.79	8.67 ± 7.87	6.93 ± 7.75	0.497

* Continuous variables were reported as mean ± standard deviation (SD). TMP-SMX, Trimethoprim–sulfamethoxazole.

**Table 4:** Univariable and multivariable logistic regression analysis of laboratory variables linked to 30-day mortality in hospitalized patients with *S. maltophilia* infections.

	Univariable Logistic Regression		Multivariable Logistic Regression	
	Odds Ratio (95% CI)	*p*-Value	Adjusted Odds Ratio (95% CI)	*p*-Value
White blood cells	1.027 (0.998–1.058)	0.070	1.133 (1.052–1.220)	0.001
Neutrophils (%)	1.045 (1.025–1.065)	<0.001	1.036 (1.003–1.070)	0.034
Hemoglobin	0.745 (0.654–0.849)	<0.001	0.679 (0.510–0.903)	0.008
Platelets	0.994 (0.993–0.996)	<0.001	0.994 (0.990–0.997)	0.022
Prothrombin time	1.087 (1.043–1.134)	<0.001	1.086 (1.001–1.179)	0.061
Partial thromboplastin time	1.018 (1.005–1.031)	0.005	1.006 (0.987–1.026)	0.256
C-reactive protein	1.041 (1.006–1.077)	0.002	1.010 (0.963–1059)	0.529
Total Protein	0.612 (0.489–0.765)	<0.001	0.887 (0.713–1.104)	0.339
Blood urea nitrogen	1.018 (1.010–1.025)	<0.001	1.011 (0.997–1.024)	0.111

**Table 5:** Univariable and multivariable logistic regression of identified risk factors linked to 30-day mortality in hospitalized patients with *S. maltophilia* infections.

	Univariable Logistic Regression		Multivariable Logistic Regression	
Identified Risk Factors	Odds Ratio (95% CI)	*p*-Value	Adjusted Odds Ratio (95% CI)	*p*-Value
Renal failure	1.740 (1.084–2.794)	0.022	1.640 (0.991–2.713)	0.054
Hospital-acquired	4.313 (1.288–14.446)	0.018	2.479 (0.699–8.793)	0.160
Ventilator-associated pneumonia	2.019 (1.305–3.123)	0.002	0.522 (0.240–1.138)	0.102
Previous antibiotics	1.537 (1.010–2.337)	0.045	1.187 (0.753–1.871)	0.460
ICU admission	3.527 (2.300–5.408)	<0.001	3.484 (1.466–8.276)	0.005
Central venous catheter	2.629 (1.660–4.164)	<0.001	1.480 (0.882–2.484)	0.137
Mechanical ventilation	2.963 (1940–4.532)	<0.001	1.303 (0.465–3.652)	0.615
Levofloxacin monotherapy	1.272 (0.679–2.383)	0.452	1.197 (0.616–2.326)	0.597

## Data Availability

The original contributions presented in this study are included in the article. Further inquiries can be directed to the corresponding author.

## Abbreviations

APACHE	Acute Physiological Assessment and Chronic Health Evaluation
aPTT	Activated partial thromboplastin time
AST	Antimicrobial susceptibility testing
BSI	bloodstream infections
CF	Cystic fibrosis
CLSI	Clinical and Laboratory Standards Institute
DM	diabetes mellitus
HAP	Hospital-acquired pneumonia
ICU	intensive care unit
PT	prothrombin time
SOFA	Sequential Organ Failure Assessment
TMP-SMX	Trimethoprim–sulfamethoxazole
UTIs	urinary tract infections
VAP	Ventilator-associated pneumonia
